# *Lacticaseibacillus rhamnosus* SC07 Alleviates Liver Oxidative Damage Association With PPAR Signaling and Immunometabolic Reprogramming in Female Mice With Lipid Disorders

**DOI:** 10.1002/fsn3.72575

**Published:** 2026-11-08

**Authors:** Linxiu Wu, Tiantian Li, Shumin Ye, Peng Chen, Rongkang Hu

**Affiliations:** ^1^ Translational Medicine Center, The Second Affiliated Hospital of Wannan Medical College Wuhu Anhui PR China; ^2^ Fuzhou Institute of Oceanography, Minjiang University Fuzhou Fujian PR China; ^3^ Anhui Provincial Key Laboratory of Molecular Enzymology and Mechanism of Major Metabolic Diseases, College of Life Sciences, Anhui Normal University Wuhu Anhui PR China; ^4^ Department of Hepatobiliary Surgery The Second Affiliated Hospital of Wannan Medical College Wuhu Anhui PR China

**Keywords:** antioxidant, immune cell infiltration, lipid accumulation, oxidative stress, probiotic strain, transcriptome

## Abstract

Liver oxidative damage, often stemming from lipid metabolic dysregulation, represents a critical juncture in liver pathophysiology. This study evaluated the therapeutic potential of *Lacticaseibacillus rhamnosus* SC07 against hepatotoxicity, with a specific focus on lipid‐driven oxidative injury. The results showed that strain SC07 could tolerate simulated gastric juice and intestinal fluid, as well as effectively reduce serum lipid accumulation and increase liver antioxidant enzyme activity in mice. Transcriptome results showed that, beyond rectifying existing gene expression abnormalities, strain SC07 exhibited modulatory effects on basal gene expression patterns. A key observation was the activation of PPAR signaling through up‐regulation of *Scd1* and *Pparg*, which was associated with lipid metabolic reprogramming and concurrent immunocyte remodeling, including expansion of regulatory Treg cells and activated natural killer cells, as well as suppression of pro‐inflammatory macrophages. This coordinated immunometabolic shift was correlated with reductions in lipid accumulation, restored cholesterol homeostasis, and enhanced antioxidant defenses, as confirmed by increased SCD1 and PPARG protein expression, ultimately alleviating lipid‐driven oxidative stress and improving liver function. These coordinated improvements position strain SC07 as a metabolic regulator capable of ameliorating oxidative hepatopathy associated with lipid disorders.

## Graphical abstract

*Lacticaseibacillus rhamnosus* SC07 alleviates lipid‐driven liver oxidative damage by activating PPAR signaling to reprogram lipid metabolism and remodel immunocyte populations, thereby enhancing antioxidant defenses and restoring liver function.

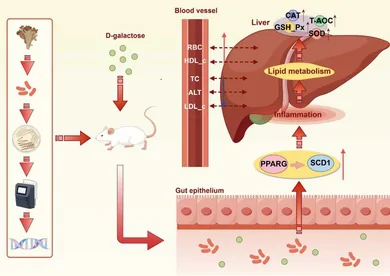

## 1 Introduction

Liver oxidative damage is widely recognized as a key pathophysiological mechanism underlying both acute and chronic liver diseases (Seen 2021). A growing number of studies are investigating potential drugs to prevent liver oxidative damage. However, their safety and efficacy in patients with chronic liver diseases remain unclear (Adelina 2022; Chen et al. 2023). Therefore, identifying complementary and alternative therapeutic agents is crucial for treating this condition.

The growing interest in bioecological and nutritional approaches to disease prevention and management underscores the therapeutic potential of probiotics across a wide range of disorders (Campaniello et al. 2023). For instance, *Lacticaseibacillus paracasei* has demonstrated antioxidant activity that mitigates D‐galactose‐induced organ damage in experimental models (Cheng et al. 2021; Kumar et al. 2022). Lactic acid bacteria represent the earliest and most extensively utilized microbial interventions in human microecological modulation, with no adverse effects documented in studies to date. Current evidence increasingly supports the prebiotic potential of select lactic acid bacteria, including *Lacticaseibacillus* species (Zhang et al. 2025). *L. rhamnosus* is among the most researched *Lacticaseibacillus*. Although *L. rhamnosus* has a long‐term safety history, its therapeutic potential against liver oxidative damage remains underinvestigated. To date, only limited evidence links *L. rhamnosus* to attenuation of liver oxidative damage through modulation of gut microbiota diversity (Jin et al. 2022), protection of intestinal cells (Zhu et al. 2022), and regulation of serum lipid profiles and hepatic enzyme levels (Aghaei et al. 2023). Moreover, exopolysaccharides isolated from *L. rhamnosus* have been shown to possess antioxidant and anti‐inflammatory activities (Zhu et al. 2022). Nevertheless, the potential intervention mechanisms and pathways through which *L. rhamnosus* alleviates liver oxidative damage remain to be fully elucidated.

A potential *L. rhamnosus* strain, designated as *L. rhamnosus* SC07, was isolated from sauerkraut. Strain SC07 exhibited remarkable tolerance in a simulated gastrointestinal environment, providing a crucial guarantee for its survival and functional performance in vivo. Additionally, the antioxidant capacity of lactic acid bacteria is generally associated with mechanisms such as free radical scavenging, regulation of oxidative stress signaling pathways, and secretion of antioxidant enzymes (Gamallat et al. 2018; Peng et al. 2022; Wu et al. 2022). The high survival rate of strain SC07 in the simulated gastrointestinal environment suggests that its antioxidant‐related enzyme systems and metabolic pathways may remain functionally stable, thereby establishing a biologically plausible foundation for investigating its antioxidant effects in vivo. However, the antioxidant activity and nutritional benefits of *L. rhamnosus* SC07, particularly its preventative and therapeutic benefits on liver oxidative damage and the underlying molecular mechanisms, remain insufficiently characterized.

Chronic D‐galactose administration has been demonstrated to promote hepatocyte apoptosis and, ultimately, oxidative and inflammatory damage in mice livers (Ma et al. 2023). We used this model to test *L. rhamnosus* SC07's ability to prevent and treat liver oxidative damage, and liver RNA‐seq and functional enrichment analysis identified the underlying molecular pathways.

## 2 Materials and Methods

### 2.1 Strain and Materials

The *L. rhamnosus* SC07 used in this study was isolated and preserved from sauerkraut by biotechnology laboratory of Anhui Normal University. Pepsin and trypsin (from Guangzhou Saiguo Bio‐Technology Co. Ltd., China) were used to prepare simulated artificial gastric juice and intestinal fluid, respectively. Preparation of these media followed established protocols (Sabater et al. 2000). 0.35 g pepsin was dissolved in 100 mL of 0.2% sterile normal saline, the pH was adjusted to 3.0 with hydrochloric acid and the artificial gastric juice was prepared via filtration and sterilizing. The artificial intestinal fluid was prepared by dissolving 0.1 g trypsin and 0.3 g bile salt in 100 mL of 0.85% normal saline, bringing the pH to 8.0 with sodium hydroxide, and filtering it. Unless otherwise specified, all other chemicals used in this study were analytical grade.

### 2.2 Biochemical Characteristics and Sequence Analysis

The freeze‐dried powder of *L. rhamnosus* SC07 was prepared by culturing the bacterium to its late logarithmic phase, followed by centrifugation at 6000 × g for 15 min at 4°C to harvest the cells. The bacterial pellet was then resuspended in sterile 10% (w/v) reconstituted skim milk as lyoprotectant. The suspension was pre‐frozen at −80°C overnight and subsequently lyophilized under vacuum (0.04 mbar, condenser temperature −55°C) for 24 h. This lyophilization protocol typically yields a viable cell concentration of approximately 10^8^ CFU g^−1^. The freeze‐dried powder of *L. rhamnosus* SC07 was added to DeMan‐Rogosa‐Sharpe (MRS) medium to achieve an initial bacterial concentration of approximately 10^5^ CFU mL^−1^, and cultured at 37°C for 48 h to obtain fresh active cultures for subsequent assays and animal experiments. After Gram staining, strain morphology was observed by microscope. Physiological and biochemical characteristics of the test strain were determined after 24 h of cultivation (Şanlıbaba and Uymaz Tezel 2024). The 16S rDNA gene of the tested strain was amplified by PCR using universal primers 27F (5′‐AGAGTTTGATCCTGGCTCAG‐3′) and 1492R (5′‐TACGGCTACCTTGTTACGACTT‐3′). The database searches of sequence data were performed using the BLASTn program in NCBI (https://blast.ncbi.nlm.nih.gov/Blast.cgi). The alignment of the obtained sequences was conducted using Clustal X version 1.81 (Thompson et al. 1994). A bootstrap consensus tree was constructed using the neighbor‐joining method packaged in MEGA version 11 (Tamura et al. 2013). Antibiotic resistance determinants were systematically identified using the Resistance Gene Identifier (RGI) tool against the Comprehensive Antibiotic Resistance Database (CARD), while putative virulence factors were comprehensively annotated using the VFanalyzer pipeline integrated into the Virulence Factor Database (VFDB) (Alcock et al. 2023; Zhou et al. 2025).

### 2.3 Tolerance Test of Strain SC07 in Artificial Gastric Juice and Intestinal Fluid

Acid resistance (pH 1–5) (Leite et al. 2015) and bile salt tolerance (0%–0.5% w/v) (Arnold et al. 2018) of *L. rhamnosus* SC07 were assessed in MRS broth at 37°C. Acid challenges lasted 6 h and bile salt exposure lasted 8 h; all assays used a 4% (v/v) inoculum. To determine the tolerance of the tested strain to artificial gastric juice (Chinese Pharmacopoeia Commission 2020), 1 mL of bacterial suspension was added to 9 mL of artificial gastric juice, and the mixture was fully mixed and cultured at 37°C for 3 h. To determine the tolerance of the tested strain to artificial intestinal fluid, 1 mL of bacterial suspension treated with artificial gastric juice for 3 h was added to 9 mL of artificial intestinal fluid and cultured at 37°C for 8 h. The plate counting of each group was performed. Each value was derived from three independent replicate experiments.

### 2.4 Animal Experiment

A total of 40 female KM mice weighing 20 ± 2 g were housed at the Second Affiliated Hospital of Wannan Medical College, Wuhu, China. All mice were bred under controlled environment (temperature of 24.0°C ± 0.5°C, humidity of 55.0% ± 5.0%) with a light/dark cycle. All mice had free access to water and standard pelleted chow with a gross energy content of 3.42 kcal g^−1^, consisting of 65.42% carbohydrates, 22.47% protein, and 12.11% fat. The study was conducted and approved by the Ethics Committee of the College of Life Sciences, Anhui Normal University (approval no. AHNU‐ET‐2021‐13, Date July 1, 2021), while the mice were housed and maintained at the animal facility of the Second Affiliated Hospital of Wannan Medical College under this approved protocol and the institutions' collaborative arrangement. This work has been carried out in accordance with the U.K. Animals (Scientific Procedures) Act, 1986 and associated guidelines. After 1‐week adaptation period, the mice were randomly assigned into four groups (10 mice each group), including normal control (NC) group, model control (MC) group, oral *L. rhamnosus* SC07 group and oral ascorbic acid‐positive control (VC) group. The MC, SC07 and VC group were subcutaneously injected with D‐galactose (Ahangarpour et al. 2018; Deng et al. 2023; Tarbiat et al. 2024) dissolved in normal saline (0.9%, w/v) (200 mg kg^−1^ d^−1^) once per day to initiate the liver oxidative damage model. The test strains were collected through centrifugation of 1 mL of bacterial solution (approximately 10^9^ CFU mL^−1^) and subsequently suspended in 0.5 mL of normal saline for intragastric administration. The mice in the SC07 group and the mice in the VC group were given normal saline solution containing strain SC07 (10^9^ CFU d^−1^) (Bai et al. 2021; Jang et al. 2019; Olimpio et al. 2022) and ascorbic acid (200 mg kg^−1^ d^−1^) (He et al. 2021; Qian et al. 2018; Zhang et al. 2020) by gavage each day, respectively. The NC and MC group received daily intragastric administration of normal saline solution. One mouse in the MC group died during the experimental period, leaving nine mice in this group for the final analyses.

### 2.5 Biochemical Index Determination

From 0 to 8 weeks, the body weight was recorded weekly. All mice were sacrificed by cervical dislocation after anesthesia (45 mg/kg pentobarbital sodium) following a 12 h fast at the end of the experiment. The liver was isolated immediately, weighed, and stored at −80°C for further analysis. Blood was immediately collected from the mice retro‐orbitally. The whole blood was collected for detecting relevant indexes (Hu et al. 2025), including red blood cell count (RBC), total cholesterol (TC), high‐density lipoprotein‐cholesterol (HDL‐c), and low density lipoprotein‐cholesterol (LDL‐c), platelet count (PLT), alanine transaminase (ALT), total protein (TP), and hemoglobin (HGB), using commercial kits (Nanjing Jiancheng Bioengineering Institute, Nanjing, China). For at least 24 h, a portion of the liver was preserved in 10% neutral‐buffered formalin before being stained with hematoxylin and eosin. Following dehydration and mounting, the sections were observed under a × 10 magnification optical microscope. The remaining liver tissue was homogenized and centrifuged, and the supernatant was collected to determine liver oxidation. The content of malondialdehyde (MDA) and the activities of total antioxidant capacity (T‐AOC), superoxide dismutase (SOD), catalase (CAT), and glutathione peroxidase (GSH‐Px) in liver (Hu et al. 2024) were measured using commercial kits (Nanjing Jiancheng Bioengineering Institute, Nanjing, China).

### 2.6 Transcriptome Sequencing of Liver

Total RNA was extracted from liver tissue using RNAiso Plus (Takara, Dalian, China). RNA integrity and concentration were assessed with an Agilent Bioanalyzer 2100 system using the RNA Nano 6000 Kit (Agilent Technologies, CA, USA). RNA samples were then submitted to Novogene Co. Ltd. (China) for transcriptome sequencing. Libraries were constructed using the NEBNext Ultra RNA Library Prep Kit for Illumina (New England Biolabs, USA), with unique index adapters ligated to each sample for multiplexing. After cluster generation on an Illumina cBot System, paired‐end sequencing was performed on an Illumina HiSeq platform, generating 125/150 bp reads. Gene‐level read counts were obtained using HTSeq (v0.6.1) and normalized to FPKM (Fragments Per Kilobase per Million mapped reads). Differential expression analysis between groups was conducted using the limma and edgeR packages (v1.18.0) (Ritchie et al. 2015; Robinson et al. 2010). Genes with an adjusted *p* < 0.05 and |log_2_(fold change)| ≥ 1 were considered significantly differentially expressed (DEGs). Overlapping DEGs across comparisons were visualized with the UpSetR package (Conway et al. 2017). Functional enrichment analysis of DEGs was performed using clusterProfiler for Gene Ontology (GO) terms and Kyoto Encyclopedia of Genes and Genomes (KEGG) pathways (Yu et al. 2012), with results presented in circular, Sankey, and bubble plots. A protein–protein interaction (PPI) network was constructed using the STRING database (https://string‐db.org/) and visualized in Cytoscape (v3.8.0) (Shannon et al. 2003). Hub genes within the network were identified using the cytoHubba plugin (Chin et al. 2014) by calculating node degree, from which the top 20 genes were selected as key candidates.

### 2.7 Western Blotting Analysis

Equal amounts of total proteins (20 μg) were subject to 10% sodium dodecyl sulfate polyacrylamide gel electrophoresis and then electrotransferred onto polyvinylidene fluoride membranes (Sangon, Shanghai, China). The membranes were blocked with 5% nonfat dried milk at room temperature for 2 h, then incubated with primary antibodies against SCD1, PPARG, and β‐ACTIN (Choijookhuu et al. 2024) (1:1000; EnoGene Biotech Co Ltd. NY, USA) at 37°C for 2 h, where β‐ACTIN is the housekeeping gene. The membranes were then incubated at 37°C for 2 h with horseradish peroxidase (HRP)‐conjugated goat anti‐mouse IgG (H + L) antibody (1:1000; Beyotime Biotechnology, Shanghai, China). Immunoreactive bands were visualized using the DAB Horseradish Peroxidase Color Development Kit (Beyotime Biotechnology, Shanghai, China). The results of western blot were analyzed by the Image J program. Eight mice were randomly selected from each group.

### 2.8 Immunoinfiltration Analysis

Immunoinfiltration analysis of the expression data was performed using our RNA‐seq database of mice liver by the CIBERSORT algorithm to analyze the immune cells of each group (Tang et al. 2023), and used heatmap and lollipop chart to visualize immune cell expression and distribution across all sample groups. In addition, we utilized ANOVA to assess the difference in the proportion of immune cells. The results of *p* < 0.05 were considered significant. The Spearman correlation analysis was conducted to assess the correlation between key genes implicated in liver damage and immune cell infiltration, with results visualized using the ggplot2 package.

### 2.9 Statistical Analyses

The body weight, liver index, biochemical index, and liver biochemical function data were expressed as means ± SD. GraphPad 9.0.2 software was used to check the assumptions for ANOVA. The normality of distribution was confirmed by the D'Agostino‐Pearson test, and the homogeneity of variances was confirmed by the Brown‐Forsythe test. Subsequently, the statistical significance was determined using one‐way analysis of variance (ANOVA) followed by Dunnett's test, with the MC group as the reference. A significant difference was represented as “*”, and a value of *p < 0.05 or **p < 0.01 was regarded as significant difference or extremely significant difference, respectively.

## 3 Results

### 3.1 The Test Strain Was Identified as *L. rhamnosus*

The physiological and biochemical characteristics of *Lacticaseibacillus* strain SC07 were determined, as summarized in Table S1. The tested strain fermented arabinose, cellobiose, levulose, galactose, glucose, lactose, maltose, esculin, mannose, mannitol, sorbitol, sucrose, salicin, and trehalose, but not meliobiose, raffinose, or xylose. It was non‐motile, and tests for gelatin liquefaction, nitrate reduction, and benzidine production were negative. These traits were consistent with those described for *L. rhamnosus* in “Bergey's manual of determinative bacteriology” (Buchanan and Gibbons 1958). As shown in Figure S1, the tested strain was Gram‐positive. Phylogenetic analysis based on 16S rDNA sequences revealed 97%–100% similarity among related strains, and BLASTn analysis confirmed its identification as *L. rhamnosus* . In addition, neither transferable antibiotic resistance genes nor characterized virulence factor genes were found, demonstrating the genetic safety of the strain for application as a probiotic and functional food ingredient (Table S2, Figure S2).

### 3.2 *L. rhamnosus* SC07 Tolerated Artificial Gastric Juice and Intestinal Fluid

As shown in Table 1, SC07 grew slowly in the MRS medium with pH 3, but its growth accelerated significantly at pH 4 and above. After incubation with artificial gastric juice (pH 3.0) for 3 h, the viable count of SC07 decreased from 7.69 to 6.94 lg (CFU/mL), corresponding to a survival rate of 90.25%. The strain exhibited good tolerance to bile salts, growing well at concentrations of 0.05% and 0.1%. At a higher concentration of 0.3%, growth was observed within 4 h but was inhibited with prolonged incubation. Following treatment with artificial intestinal fluid, the bacterial count decreased from 6.94 to 5.38 (CFU/mL) after 4 h (survival rate: 77.52%) and further to 4.84 lg (CFU/mL) after 8 h (survival rate: 69.74%).

**TABLE 1:** Resistance of *Lacticaseibacillus rhamnosus* strain SC07 to different environments.

Time (h)	OD_600_					Bacterial count in artificial gastric juice [lg (CFU/mL)]	Survival rate (%)
	pH 1	pH 2	pH 3	pH 4	pH 5		
0	0.142 ± 0.002	0.142 ± 0.002	0.142 ± 0.002	0.142 ± 0.002	0.142 ± 0.002	7.69 ± 0.04	
3	0.132 ± 0.006	0.134 ± 0.011	0.161 ± 0.008	0.379 ± 0.023	0.506 ± 0.028	6.94 ± 0.13	90.25
6	0.108 ± 0.005	0.112 ± 0.009	0.382 ± 0.024	1.294 ± 0.087	1.532 ± 0.105		

Time (h)	OD_600_					Bacterial count in artificial intestinal fluid [lg (CFU/mL)]	Survival rate (%)
	0%	0.05%	0.10%	0.30%	0.50%		
0	0.138 ± 0.002	0.138 ± 0.002	0.138 ± 0.002	0.138 ± 0.002	0.138 ± 0.002		
4	0.695 ± 0.017	0.631 ± 0.029	0.622 ± 0.024	0.466 ± 0.013	0.125 ± 0.006	5.38 ± 0.08	77.52
8	1.733 ± 0.084	1.283 ± 0.056	1.024 ± 0.019	0.382 ± 0.018	0.111 ± 0.005	4.84 ± 0.06	69.74

*Note:* Each value is expressed as mean ± SD.

### 3.3 *L. rhamnosus* SC07 Effectively Protected Liver Function

The experimental design to evaluate the preventive and therapeutic effects of *L. rhamnosus* SC07 on liver oxidative damage is shown in Figure 1A. During the first 2 weeks, body weights in the MC and SC07 groups were slightly lower than in the NC group. While no difference was observed between the MC and SC07 groups for the initial 4 weeks, the weight gain of the MC group subsequently became progressively lower than that of the SC07 group (Figure 1B). As shown in Figure 1C, the liver index (calculated as liver weight/body weight) was significantly increased in the MC group compared to the NC group, and this increase was markedly attenuated by SC07 treatment (*p* < 0.01). Hematological analysis revealed impaired liver function in the MC group, characterized by significantly elevated levels of TC, LDL‐c, and ALT, and reduced levels of RBC, HDL‐c, PLT, and HGB compared to the NC group (Figure 1D). In contrast, SC07 administration significantly increased RBC and HDL‐c levels (*p* < 0.05), and decreased TC, LDL‐c, and ALT levels (*p* < 0.01) relative to the MC group. No significant effects of SC07 treatment were observed on PLT, HGB, or TP levels in D‐galactose‐treated mice. These modulations of key lipid metabolism markers by SC07 suggest its potential to mitigate liver oxidative damage by reducing lipid accumulation and restoring cholesterol homeostasis.

**FIGURE 1:**
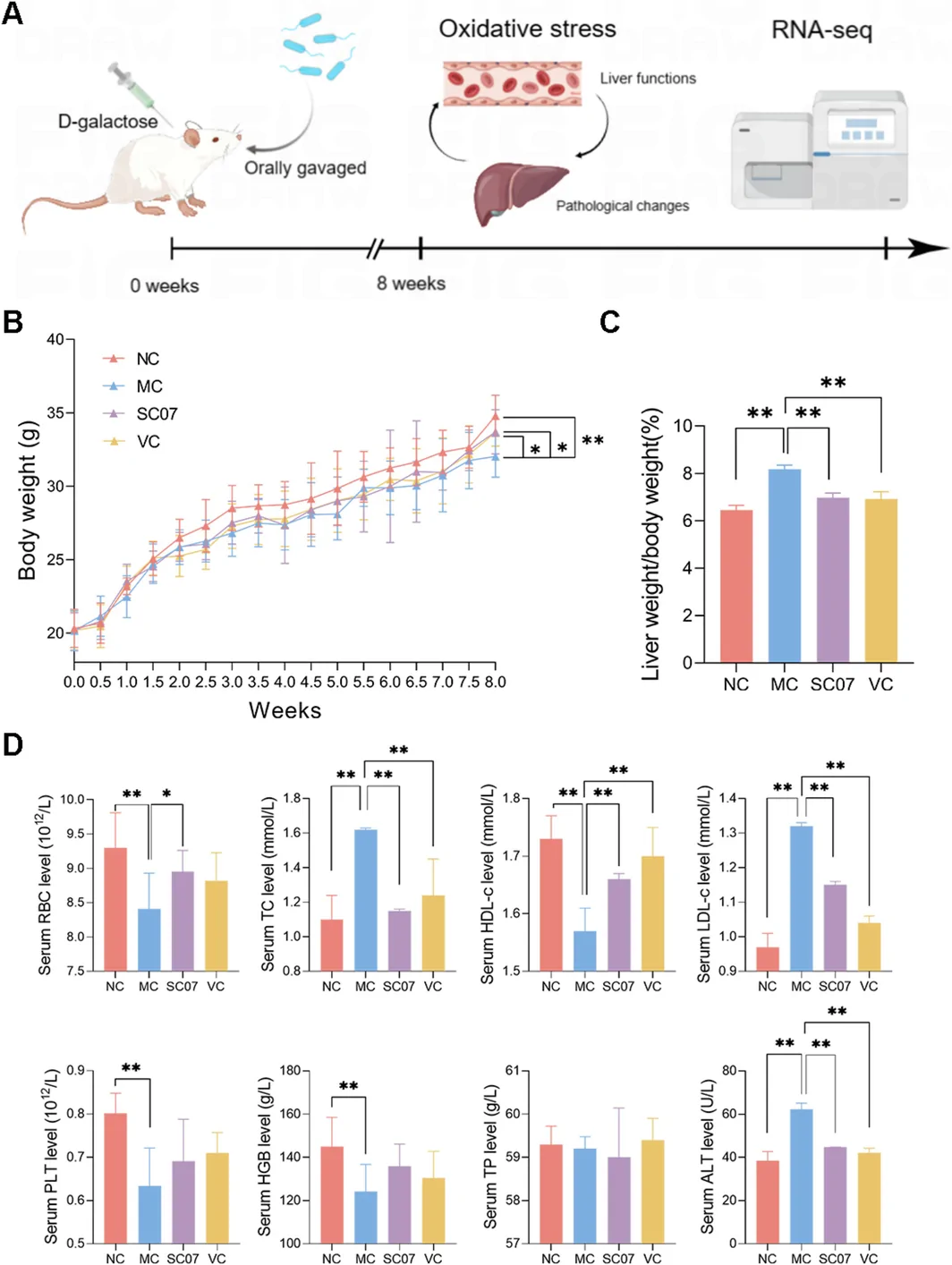
Effects of *Lacticaseibacillus rhamnosus* SC07 on the liver functions of D‐galactose‐treated mice. (A) Experimental scheme and workflow diagram. (B) Body weight of mice in different groups. (C) Liver index of mice in different groups. (D) Biochemical index of mice in different groups. Normal control group (NC), model control group (MC), oral *L. rhamnosus* SC07 group (SC07), and oral ascorbic acid‐positive control group (VC). The NC, SC07, and VC groups were each compared with the MC group. Statistical significance: *p < 0.05 or **p < 0.01. Data are presented as mean ± SD. Sample sizes: *N* = 10 for blank, experimental, and positive control groups; *n* = 9 for the model control group (one mouse died at week 1.5).

### 3.4 *L. rhamnosus* SC07 Alleviated Liver Histopathology and Oxidative Stress

Liver histology revealed intact liver architecture in the NC group, with hepatocytes neatly arranged around the central vein (Figure 2A). In contrast, D‐galactose administration (MC group) induced marked pathological alterations, including edema, vacuolation, and dilated, congestive central veins. Treatment with *L. rhamnosus* SC07 or ascorbic acid effectively mitigated these alterations and preserved liver architecture. Consistent with the histopathological findings, oxidative stress markers were significantly dysregulated in the MC group compared to the NC group (Figure 2B). Activities of SOD, GSH‐Px, T‐AOC, and CAT were significantly decreased (*p* < 0.05), while MDA content was elevated (*p* < 0.05). Supplementation with strain SC07 notably reversed these changes: it increased the activities of SOD, GSH‐Px, and CAT, with a particularly pronounced elevation in T‐AOC activity (*p* < 0.01) relative to the MC group. This restoration of antioxidant defenses suggests that SC07 ameliorated the liver oxidative stress linked to lipid metabolic disorders.

**FIGURE 2:**
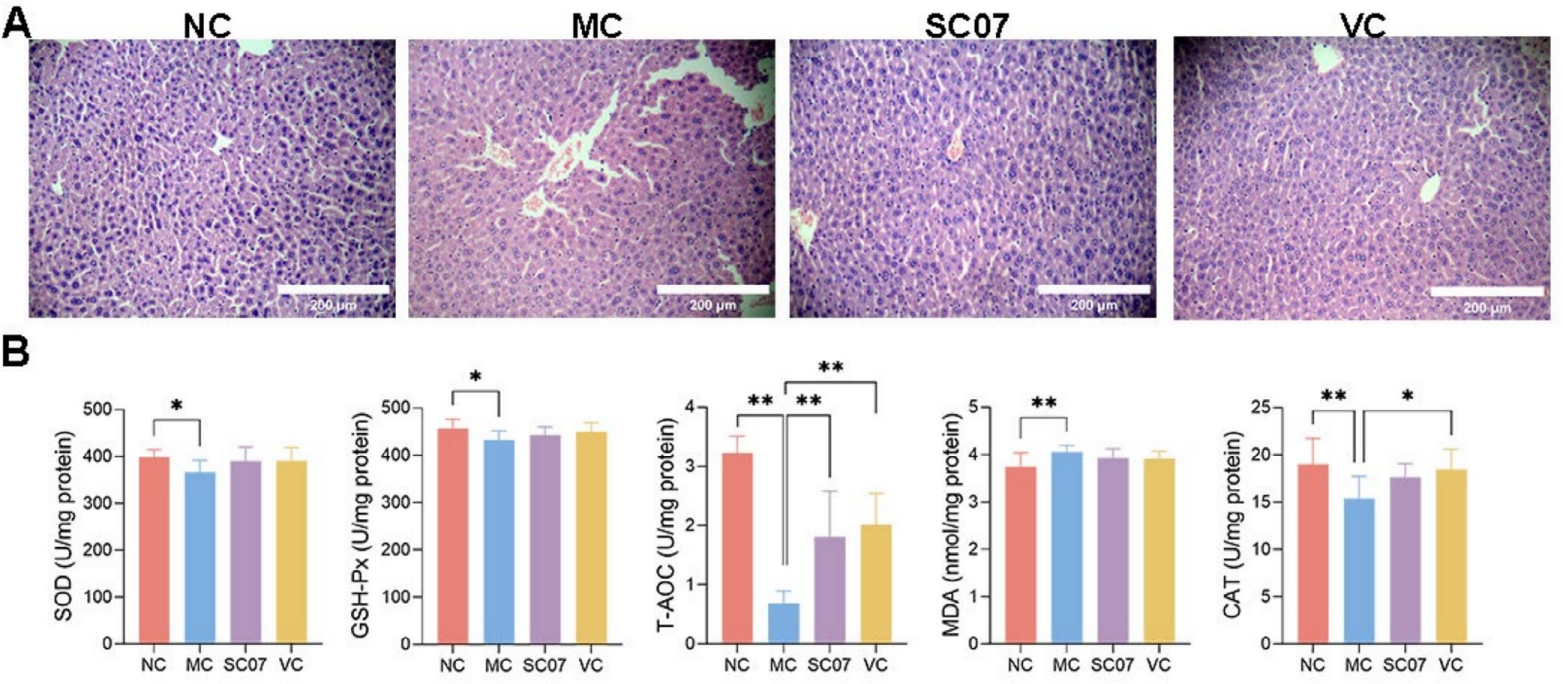
Effects of *Lacticaseibacillus rhamnosus* SC07 on the pathological changes and oxidative stress of the liver in D‐galactose‐treated mice. (A) Histopathological characterization of the experimental mice livers (H&E stain, ×10 magnification), scale bar = 200 μm. (B) Effects of *L. rhamnosus* SC07 on the levels of superoxide dismutase (SOD), catalase (CAT), glutathione peroxidase (GSH‐Px), total antioxidant capacity (T‐AOC) and malondialdehyde (MDA) in the experimental mice livers. Normal control group (NC), model control group (MC), oral *L. rhamnosus* SC07 group (SC07) and oral ascorbic acid‐positive control group (VC). The NC, SC07, and VC groups were each compared with the MC group. Statistical significance: *p < 0.05 or **p < 0.01. Data are presented as mean ± SD. Sample sizes: *N* = 10 for blank, experimental, and positive control groups; *n* = 9 for the model control group (one mouse died at week 1.5).

### 3.5 *L. rhamnosus* SC07 Modulated Liver Gene Expression

To elucidate the molecular mechanisms underlying its protective effects, we performed transcriptomic sequencing on liver tissues. Compared to the NC group, D‐galactose exposure (MC group) induced differential expression of 236 genes (176 up‐ and 60 down‐regulated) (Figure S3A). SC07 treatment significantly altered the expression of 234 genes relative to the MC group (191 down‐ and 43 up‐regulated) (Figure S3B). Among these, 226 genes were specifically associated with SC07 intervention. This intervention demonstrated restorative capabilities by normalizing the expression of 90 (cyan) out of 176 up‐regulated genes and 12 (cyan) out of 60 down‐regulated genes that were initially dysregulated in the MC group, indicating a reversal of D‐galactose‐induced transcriptional disturbances (Figure S3C). Intriguingly, SC07 also modulated the expression of 124 genes (gray cluster) that were not differentially expressed in the MC versus NC comparison, suggesting an effect beyond mere correction of pathology. This dual regulatory capacity, encompassing both correcting disease‐associated gene expression and modulating basal transcriptional programs, highlights a multifaceted mechanism by which SC07 may ameliorate oxidative liver damage associated with lipid metabolic disorders.

### 3.6 Functional Enrichment Analysis Identifies Key Genes and Pathways in SC07‐Mediated Protection

To explore the functions of the 102 genes that exhibited differential regulation in the MC_vs._NC group and the SC07_vs._MC group (Group 1), as well as the 124 genes that exhibited regulation in the SC07_vs._MC group but not in the MC_vs._NC group (Group 2), we performed GO and KEGG analyses. GO analysis revealed enrichment in processes related to metabolism, biosynthesis, oxidation, cholesterol homeostasis, and liver development (Figure 3A). From these, 79 genes were prioritized as candidate liver function regulators (Figure 3B). Group 1 contained 37 genes; key genes (*Nr1d1, Cyp4a12*b, *Corin*, *Hbb_bs*, and *Hba_a*) down‐regulated by D‐galactose were up‐regulated by SC07, while others showed the opposite pattern. Furthermore, Group 2 contained 42 genes, of which 10 genes, including *Scd1*, *Hba_a1*, and *Ddah1* showed up in the SC07_vs._MC group, and 32 genes such as *Hhex*, *Pcsk9*, and *Cyp2b10* showed down in the SC07_vs._MC group. Notably, in Group 2, genes that were differentially expressed in the SC07_vs._MC group were not differentially expressed in the MC_vs._NC group.

**FIGURE 3:**
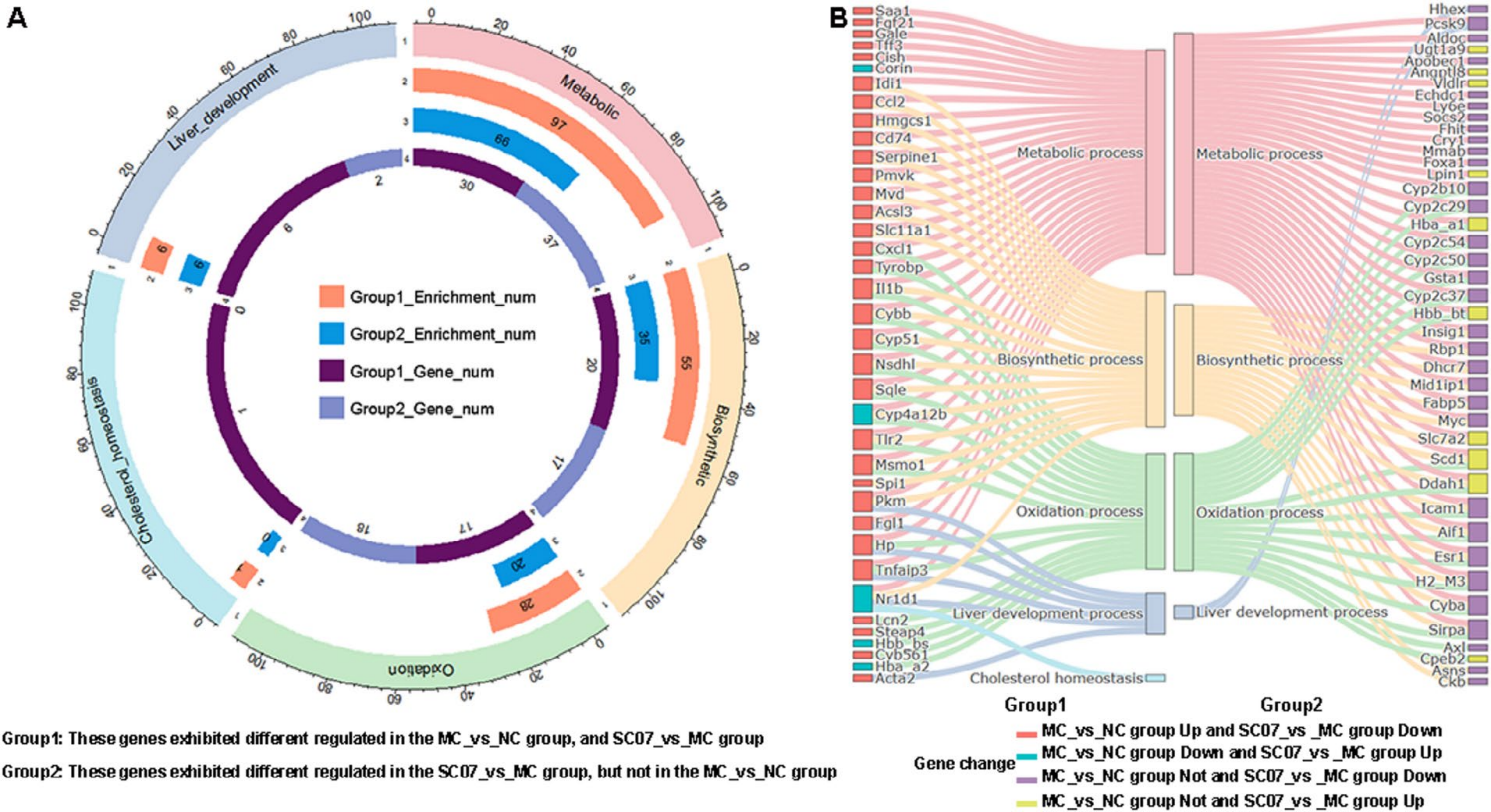
Gene Ontology (GO) enrichment analysis of differential gene expression. (A) Circle diagram of gene biological function by GO. (B) Sankey diagram of linked differential gene expression and biological function enrichment result. Normal control group (NC), model control group (MC), and oral *Lacticaseibacillus rhamnosus* SC07 group (SC07). For liver transcriptome analysis, *n* = 8 mice per group.

To clarify the regulatory mechanism of differentially expressed genes in liver oxidative damage, this study focused on analyzing the signaling pathways related to lipid homeostasis through KEGG pathway analysis (Figure 4). A total of 32 genes from both groups were implicated in PPAR, Chemokine, IL‐17, NF‐kappa B, TNF, NOD‐like receptor and toll‐like receptor signaling pathway. In Group 1, SC07 down‐regulated 18 D‐galactose‐induced genes (e.g., *Cxcl1*, *Lcn2* and *Gadd45g*) and up‐regulated *Cyp4a12b*. In Group 2, SC07 modulated 13 genes with stable expression in the MC_vs._NC comparison, down‐regulating 10 and up‐regulating 3 (*Pparg*, *Scd1* and *Plin4*). Notably, the three up‐regulated genes in Group 2 (*Pparg*, *Scd1* and *Plin4*) were significantly enriched in the PPAR signaling pathway, which coordinately regulates fatty acid oxidation, lipid droplet formation, and storage (Sabater et al. 2000).

**FIGURE 4:**
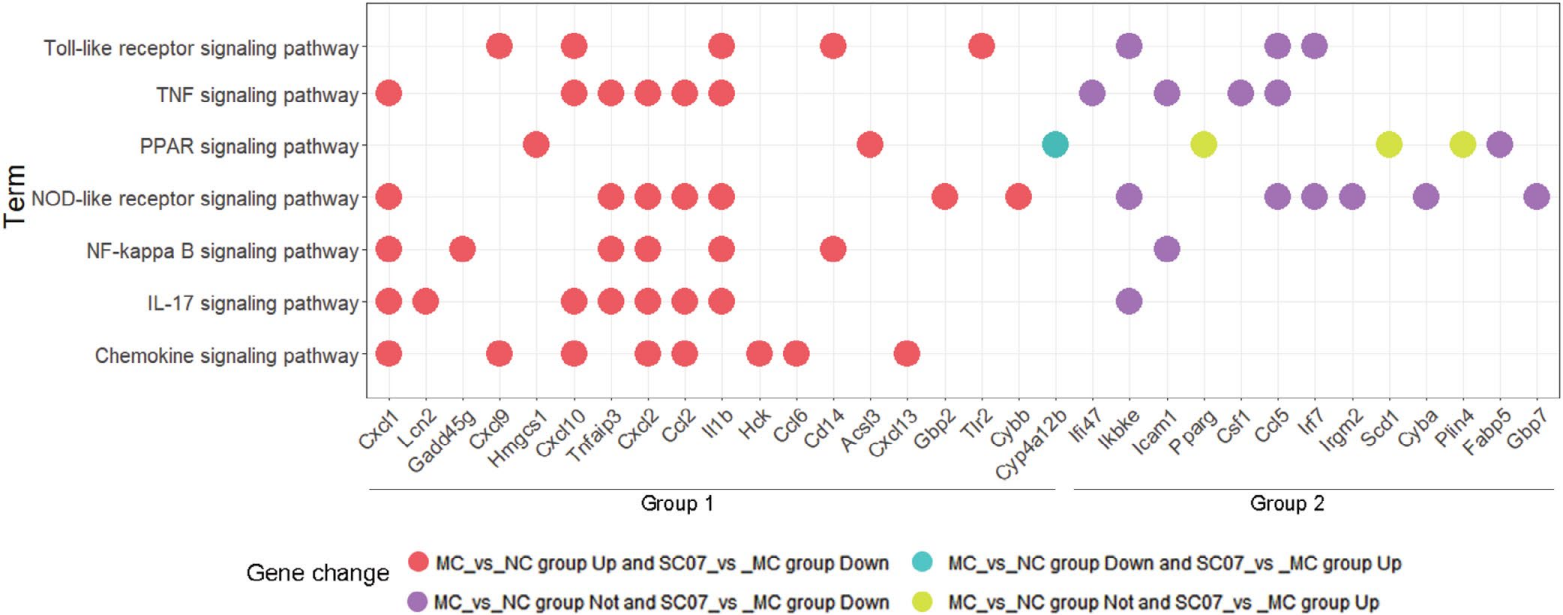
Kyoto Encyclopedia of Genes and Genomes (KEGG) pathway enrichment analysis of differentially expressed genes. Normal control group (NC), model control group (MC), and oral *Lacticaseibacillus rhamnosus* SC07 group (SC07). For liver transcriptome analysis, *n* = 8 mice per group.

### 3.7 *Pparg* and *Scd1* Are Key Mediators in SC07‐Mediated Alleviation of Liver Oxidative Damage

A PPI network was constructed by integrating the 79 GO‐selected and 32 KEGG‐enriched genes, after removing duplicates, using the STRING database. Hub genes within this network were identified in Cytoscape with the cytoHubba plugin using the degree algorithm (Figure 5A). Based on centrality degree values, the top 20 hub genes, including *Il1b*, *Ccl2*, *Tlr2*, *Pparg*, *Ccl5*, *Icam1*, *Cxcl10*, *Csf1*, *Cybb*, *Cxcl1*, *Cd14*, *Irf7*, *Aif1*, *Lcn2*, *Cd74*, *Cxcl9*, *Scd1*, *Acta2*, *Serpine1*, and *Cxcl2*, were considered potential biomarkers for mitigating liver oxidative damage. Pearson correlation analysis between these hub genes and key metabolic parameters revealed distinct association patterns (Figure 5B). Notably, only *Scd1* and *Pparg* were found to be positively correlated with beneficial metabolic and hematological parameters (T‐AOC, RBC, HDL‐c, and body weight), and negatively correlated with adverse lipid profiles LDL_c, TC, ALT, and liver index (Figure 5B). Next, we validated SCD1 and PPARG protein expression in all groups by performing western blotting. As shown in Figure S4 and Figure 5C, SC07 treatment significantly up‐regulated both SCD1 and PPARG protein levels compared to the MC group, supporting their potential central role in lipid homeostasis. This functional validation, combined with their prior identification within the PPAR signaling pathway (Figure 4), underscores that SC07 alleviates D‐galactose‐induced liver oxidative damage, at least in part, by modulating this key lipid metabolic pathway via *Scd1* and *Pparg*.

**FIGURE 5:**
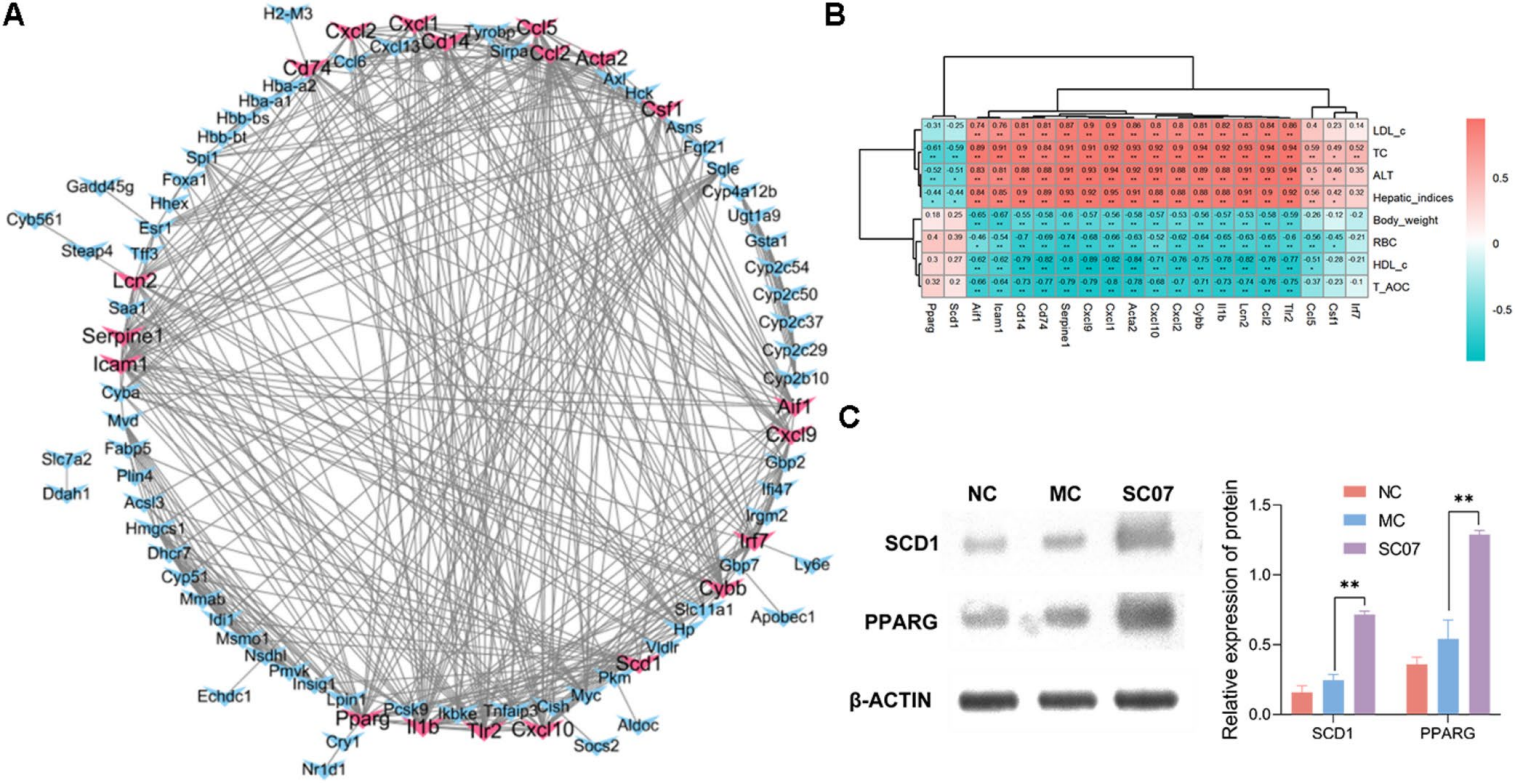
Identification of the key genes involved in alleviating liver oxidative damage following *Lacticaseibacillus rhamnosus* SC07 treatment. (A) Protein–protein interaction network construction and hub genes identification based on STRING and Cytoscape database analyses. (B) A heatmap depicted the correlation between the biochemical indices and the hub genes in the liver. (C) Western blot analysis of SCD1 and PPARG protein expression levels. The NC and SC07 groups were each compared with the MC group. Statistical significance: *p < 0.05 or **p < 0.01. For liver transcriptome analysis, *n* = 8 mice per group.

### 3.8 *L. rhamnosus* SC07 Modulates Immune Cell Infiltration via the PPAR Pathway

Given the close link between lipid metabolism, inflammation, and immune cell profiles (Xu et al. 2024), we hypothesized that *Scd1* and *Pparg* might influence liver function through immune modulation. Analysis revealed significant differences in immune cell infiltration among groups, except for Mast cells, T cells CD8 memory, and Th1 cells (Figure 6A,B). Compared to the NC group, which showed higher infiltration of Plasma cells, T cells CD4 naive, M2 macrophage, Th17 cells, and others, the MC group exhibited elevated levels of NK resting, T cells CD8 naive, DC immature, T cells CD4 memory, GammaDelta T cells, M0 macrophage and M1 macrophage (*p* < 0.05). Interestingly, we found that cells with high degree of immune infiltration in the NC and MC groups were not enriched in the SC07 group. The SC07 group displayed a distinct immune profile, characterized by higher infiltration of T cells CD4 follicular, activated NK cells, Neutrophil cells, B cells memory, Treg cells and Monocyte. Correlation analysis further linked the key genes (*Scd1* and *Pparg*) to this immune remodeling (Figure 6C). Both *Scd1* and *Pparg* were positively correlated with Treg cells, activated NK cells, Neutrophil cells and Monocyte (*p* < 0.05), but negatively correlated with T cells CD4 memory, NK resting and DC immature (*p* < 0.05). These findings suggest that *Scd1* and *Pparg*, as core regulatory factors of the PPAR signaling pathway, contribute to the lipid metabolic reprogramming concomitant with immune cell population remodeling, which is linked to attenuated liver oxidative damage in mice.

**FIGURE 6:**
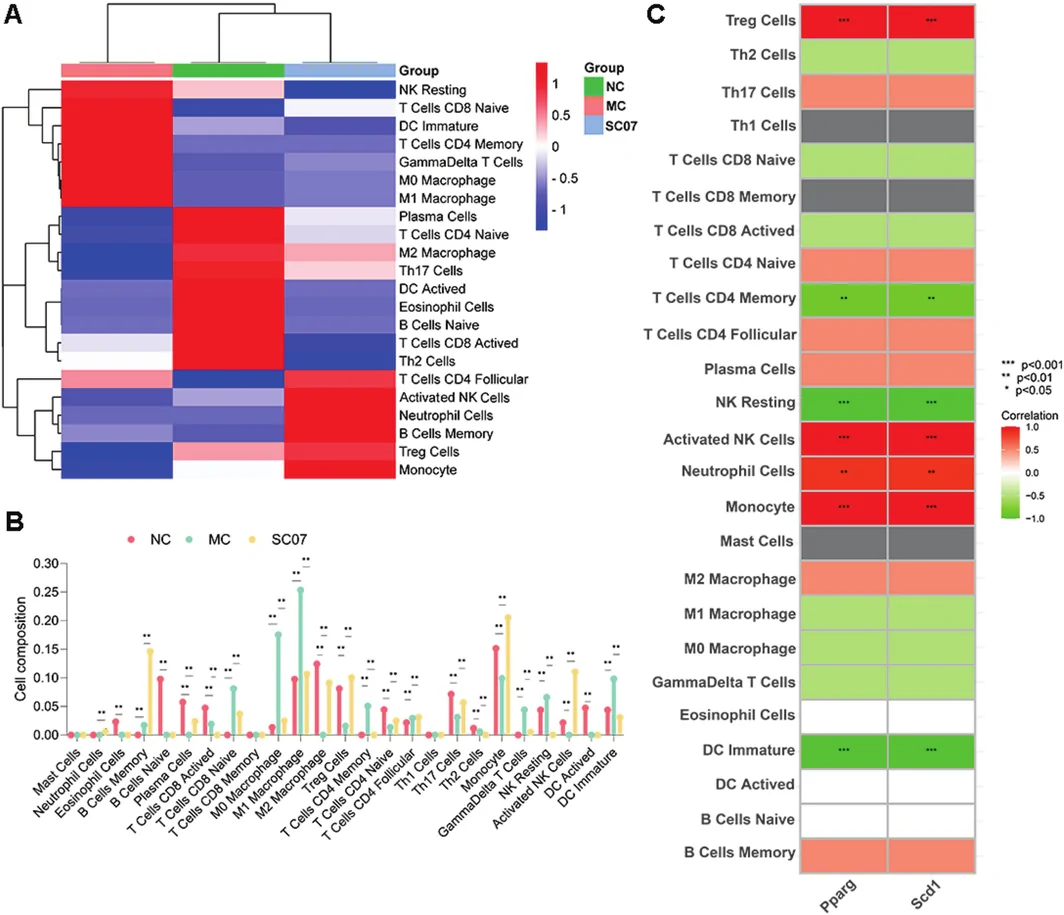
Analysis of immune cell infiltration. (A) Distribution of the immune cells in all groups. (B) Analysis of differential expression of the immune cells between groups. (C) Correlation of key genes with immune cell infiltration in liver. Normal control group (NC), model control group (MC) and oral *Lacticaseibacillus rhamnosus* SC07 group (SC07). The NC and SC07 groups were each compared with the MC group. Statistical significance: **p < 0.01 or ***p < 0.001. For liver transcriptome analysis, *n* = 8 mice per group.

## 4 Discussion

Probiotics are generally recognized as safe (GRAS) microorganisms and are widely used in food and pharma formulations (Duche et al. 2023). However, to exert probiotic benefits, lactobacilli must survive the harsh gastrointestinal environment, characterized by gastric acid, bile salts, and digestive enzymes. Human gastric juice has a pH of ~3.0, and food remains in the stomach for ~3 h. Gastric juice can damage bacterial cell membranes, proteins, and DNA, reducing viability (Nizer et al. 2020; Wang et al. 2018). Notably, *L. rhamnosus* SC07 exhibited high acid tolerance, with no decrease in viable counts after 3 h at pH 3.0 MRS medium. Subsequently, bile salts (≈0.3% in the small intestine) pose another challenge via osmotic stress and membrane disruption (Merritt and Donaldson 2009). Strain SC07 not only grew in the presence of 0.3% bile salts but also demonstrated high resilience in a simulated digestive tract: survival rates reached approximately 90% after 3 h in artificial gastric juice and remained near 70% following an additional 8 h in artificial intestinal fluid. This robust tolerance may be attributed to potential mechanisms such as bile salt hydrolase activity, which detoxifies bile acids (Foley et al. 2019), and protective cell wall/membrane components that resist enzymatic hydrolysis (Reis et al. 2012; Zhang et al. 2024).

*L. rhamnosus* is one of the most extensively studied probiotics globally. While its benefits for gut health are well‐established, research into its hepatoprotective effects, particularly against oxidative damage, has gained momentum only in recent years. Previous studies indicate that *L. rhamnosus* alleviates liver oxidative damage primarily by modulating blood lipid profiles and enhancing liver antioxidant enzyme activities (Aghaei et al. 2023; Jin et al. 2022; Wang et al. 2023). Beyond these established mechanisms, our study revealed a novel finding: SC07 treatment significantly increased RBC levels in mice with liver oxidative damage. Elevated RBC levels may enhance the blood's oxygen‐carrying capacity, thereby improving hepatic oxygen supply and supporting tissue repair (Fidanza and Forrester 2021). Furthermore, RBCs themselves possess intrinsic antioxidant enzymes that contribute to systemic free radical scavenging, offering an additional layer of protection against oxidative injury in the liver (Revin et al. 2019). Thus, the increase in RBCs induced by SC07 could confer hepatoprotection through both improved oxygen delivery and augmented systemic antioxidant defense.

Transcriptomic analysis revealed that SC07 alleviated liver oxidative damage by up‐regulating *Scd1* and *Pparg*, core components of the PPAR signaling pathway, thereby addressing underlying lipid metabolic disorders. SCD1 encodes stearoyl‐CoA desaturase 1, a key enzyme in monounsaturated fatty acid synthesis (e.g., palmitoleic and oleic acids) and lipid metabolism (Sun et al. 2024). PPARG encodes a nuclear receptor pivotal for adipocyte differentiation and a master regulator of lipid homeostasis, with recognized roles in liver disease pathogenesis (Wu et al. 2020). Clinically, this transcriptional reprogramming translated into improved serum lipid profiles (reduced LDL‐c, elevated HDL‐c) and fundamentally altered the liver immunometabolic microenvironment. The PPAR pathway activation by *L. rhamnosus* SC07 was associated with increased infiltration of anti‐inflammatory immune cells, including Treg cells, activated NK cells, Neutrophil cells and Monocyte, and a decrease in pro‐inflammatory subsets such as Th17 cells (Figure 6). This remodeling may critically underpin the metabolic improvement. For instance, the observed reduction in Th17 cells alongside a rise in Tregs suggests a promotion of immune tolerance (Wang et al. 2024), potentially mediated by SCD1‐derived lipid metabolites that influence T‐cell differentiation. Furthermore, the activation of NK cells in the SC07 group aligns with evidence that *Pparg* is involved in NK cell development and cytokine production (e.g., IL‐5, IL‐13) (Zhang and Young 2002). We thus hypothesize that *Pparg* up‐regulation may, directly or through downstream effectors like *Scd1*, promote NK cell activation and recruitment (Yao et al. 2017). Collectively, our findings support a model whereby SC07 modulates the PPAR pathway to concurrently rectify lipid metabolism and may reshape the liver immune landscape toward an anti‐inflammatory state, ultimately mitigating oxidative damage.

Collectively, our findings indicate that *L. rhamnosus* SC07 alleviates liver oxidative stress by activating the PPAR signaling pathway. This activation, driven by the up‐regulation of *Pparg* and *Scd1*, orchestrates a dual protective mechanism. First, it reprograms the hepatic immune microenvironment, notably reducing the infiltration of pro‐inflammatory M0 and M1 macrophages. Second, it systemically enhances antioxidant defense. PPAR activation promotes erythrocyte maturation (Fidanza and Forrester 2021), consistent with the elevated RBC counts observed (Figure 1D), thereby increasing the systemic delivery of endogenous antioxidants (e.g., SOD, CAT, and GSH‐Px) to mitigate oxidative damage in the liver (Revin et al. 2019). Of note, MDA represents a well‐established and reproducible surrogate marker for lipid peroxidation in D‐galactose‐induced hepatic oxidative damage, and our interpretation is reinforced by multiple complementary lines of evidence including antioxidant enzyme activities, serum lipid parameters, liver‐function indices and transcriptomic profiling. Even so, without detection of more specific lipid peroxidation adducts, we cannot provide definitive confirmation of the full lipid peroxidation pathway. The concomitant reduction in pro‐inflammatory cytokines further suggests a PPAR‐mediated synergy between antioxidant and anti‐inflammatory responses. Together, this dual modulation underlies the preserved hepatic histoarchitecture and normalized oxidative markers in SC07‐treated mice. A schematic summary of this proposed mechanism is presented in Figure 7.

**FIGURE 7:**
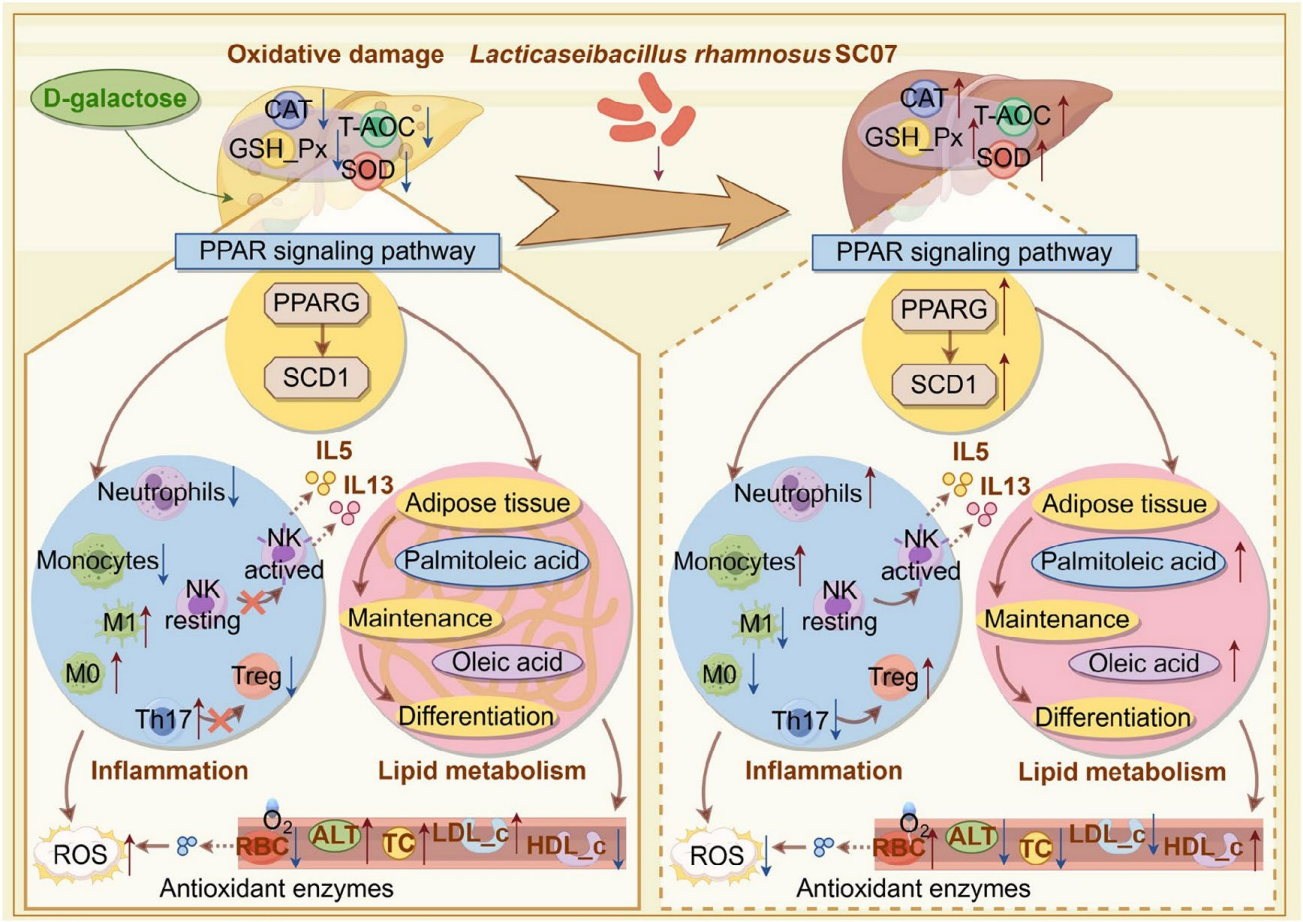
The schematic illustration of *Lacticaseibacillus rhamnosus* SC07 treatment alleviating liver oxidative damage.

Although our study reveals a robust association among SC07 treatment, activation of the PPAR signaling pathway, and specific immune cell remodeling, the exact molecular mechanism underlying the up‐regulation of *Pparg* and *Scd1* remains incompletely defined. This immunometabolic reprogramming may involve direct activation of liver or systemic PPAR signaling by bacterial components or metabolites derived from SC07. Alternatively, or additionally, SC07 may act indirectly by modulating the gut microbiota and the subsequent production of immunoregulatory metabolites (e.g., short‐chain fatty acids), which can signal through pathways including PPAR. However, short‐chain fatty acids and bile acids, which are plausible mediators linking the gut microbiota to PPAR signaling, were not measured in this study, and their quantification therefore represents an important direction for future work to directly establish the indirect, microbiota dependent arm of this mechanism. These direct and indirect mechanisms are not mutually exclusive and likely operate synergistically. While our findings establish PPAR pathway activation as a central mechanism, its safety profile warrants consideration. Synthetic PPARγ agonists are associated with adverse effects such as weight gain and fluid retention, while chronic pharmacological PPARγ activation has shown tumor promoting effects in some experimental models (Ding et al. 2025) In our study, the PPAR‐related response was accompanied by improved serum lipid profiles (reduced TC and LDL‐c, increased HDL‐c) and reduced liver injury (lower ALT), consistent with a favorable metabolic response. Modulation observed here is likely mild and host‐mediated, potentially via microbial or host‐derived factors, and differs from direct, potent, and sustained receptor activation by high‐dose synthetic ligands. Nonetheless, our 8 week, 10^9^ CFU/day regimen represents only a short‐to‐medium‐term, moderate exposure, and the long‐term consequences of chronic, high‐dose probiotic‐induced PPAR activation, including potential tumorigenic risk, remain to require dedicated long‐term safety studies.

A key limitation of this study is its exclusive use of female mice. This design was chosen to maintain model consistency, thereby providing a stable biological context for deep mechanistic investigation into PPAR‐mediated immunometabolic pathways. While this approach supports pathway discovery, it precludes direct extrapolation of the findings to males. Significant sexual dimorphism exists in liver metabolism, immune function, and oxidative stress responses, largely driven by sex hormones (Kasarinaite et al. 2023). Furthermore, the inherent antioxidant properties associated with estrogenic activity in females may have influenced the baseline model (Smiriglia et al. 2025). Although our results demonstrate SC07's potent bioactivity against oxidative damage within this female model, its therapeutic efficacy, the potential influence of the estrous cycle, and the mechanistic nuances of its action in males remain to be determined and warrant dedicated future investigation.

## 5 Conclusions

In conclusion, *L. rhamnosus* SC07 demonstrates strong gastrointestinal tolerance, surviving low pH and bile salts as well as artificial digestive fluids. In a D‐galactose‐induced murine model, SC07 administration effectively alleviated liver oxidative damage, as evidenced by the restoration of key antioxidant enzymes and serum lipid profiles. Mechanistically, transcriptomic analysis revealed that SC07 modulates the PPAR signaling pathway by up‐regulating the pivotal genes *Scd1* and *Pparg*. This activation may promote an anti‐inflammatory immune microenvironment and enhance systemic antioxidant capacity, ultimately rectifying the lipid metabolic disorders underlying liver oxidative stress. These findings highlight *L. rhamnosus* SC07 as a promising probiotic candidate for intervention in liver oxidative damage induced by lipid metabolic disorders.

## Author Contributions

**Linxiu Wu:** supervision, funding acquisition, resources, project administration, writing – original draft. **Tiantian Li:** methodology, data curation, validation, supervision, software. **Shumin Ye:** validation, software. **Peng Chen:** methodology, data curation, validation. **Rongkang Hu:** methodology, supervision, data curation, validation, investigation.

## Funding

This work was supported by the Scientific Research Plan Project of University in Anhui Province (2024AH051908), Outstanding Innovative Research Team for Molecular Enzymology and Detection in Anhui Provincial Universities (2022AH010012), Scientific Research Startup Fund Project for High‐level Talents of the Second Affiliated Hospital of Wannan Medical College (GRK202509), Wannan Medical College Young and Middle‐aged Research Fund (EYR202302), and Scientific Research Projects of the Second Affiliated Hospital of Wannan Medical College (YK2023Z02).

## Ethics Statement

All animal procedures were performed in accordance with the Guide for the Care and Use of Laboratory Animals of Anhui Normal University, and approved by the Laboratory Animal Ethics Review Committee (no. AHNU‐ET‐2021‐13).

## Conflicts of Interest

The authors declare no conflicts of interest.

## Supporting information


**Table S1:** Physiological and biochemical characteristics of *Lacticaseibacillus rhamnosus* SC07.
**Table S2:** Identification of virulence factors in the genome of the *Lacticaseibacillus rhamnosus* SC07 using the Virulence Factor Database.
**Figure S1:** 16S rDNA‐based phylogenetic tree and cell morphology (1000×) of the strain SC07.
**Figure S2:** Detection of antibiotic resistance genes in the genome of the strain SC07 by in silico screening against the Comprehensive Antibiotic Resistance Database (CARD) database.
**Figure S3:** Differential gene expression in the liver transcriptomic analysis. (A) and (B) Volcano map of differential gene expression. (C) The Venn Diagram of differential gene expression. Normal control group (NC), model control group (MC) and oral *Lacticaseibacillus rhamnosus* SC07 group (SC07). For liver transcriptome analysis, *n* = 8 mice per group.
**Figure S4:** The raw image of the full polyvinylidene fluoride membranes in Western blotting analysis. Immunoreactive bands were visualized using the DAB Horseradish Peroxidase Color Development Kit (Beyotime Biotechnology, Shanghai, China). Normal control group (NC), model control group (MC) and oral *Lacticaseibacillus rhamnosus* SC07 group (SC07).

## Data Availability

The data that support the findings of this study are available from the corresponding author upon reasonable request.
