# Supplementary material for: Lacticaseibacillus rhamnosus SC07 Alleviates Liver Oxidative Damage Association With PPAR Signaling and Immunometabolic Reprogramming in Female Mice With Lipid Disorders
Source: Food Sci Nutr. 2026 Nov 8;14(11):e72575. doi: 10.1002/fsn3.72575 (PMC13745645; doi:10.1002/fsn3.72575)
Supplement: Supplementary file 1 [file FSN3-14-e72575-s001.doc]

**Supplemental File**

***Lacticaseibacillus rhamnosus* SC07 Alleviates Liver Oxidative Damage Association with PPAR Signaling and Immunometabolic Reprogramming in Female Mice with Lipid Disorders**

Linxiu Wu^1^ | Tiantian Li^3^ | Shumin Ye^2^ | Peng Chen^4^ | Rongkang Hu^2*^

^1^Translational Medicine Center, The Second Affiliated Hospital of Wannan Medical College, Wuhu, Anhui 241000, PR China

^2^Anhui Provincial Key Laboratory of Molecular Enzymology and Mechanism of Major Metabolic Diseases, College of Life Sciences, Anhui Normal University, Wuhu 241000, Anhui, PR China

^3^Fuzhou Institute of Oceanography, Minjiang University, Fuzhou, Fujian 350108, PR China

^4^Department of Hepatobiliary Surgery, The Second Affiliated Hospital of Wannan Medical College, Wuhu, Anhui 241000, PR China

**^*^Corresponding author at:** Anhui Provincial Key Laboratory of Molecular Enzymology and Mechanism of Major Metabolic Diseases, College of Life Sciences, Anhui Normal University, Wuhu 241000, Anhui, PR China

**E-mail address:** Rongkang Hu ([Rongkang_Hu@163.com](mailto:Rongkang_Hu@163.com))

**TABLE S1 |** Physiological and biochemical characteristics of *Lacticaseibacillus rhamnosus* SC07.

| **Items** | **Results** | **Items** | **Results** |
| --- | --- | --- | --- |
| Arabinose | + | Meliobiose | - |
| Cellobiose | + | Raffinose | - |
| Levulose | + | Xylose | - |
| Galactose | + | Hydrogen sulfide test | - |
| Glucose | + | Indole test | - |
| Lactose | + | Gelatin liquidized test | - |
| Maltose | + | Nitrate test | - |
| Esculin | + | Benzidine | - |
| Mannose | + | Motility | - |
| Mannitol | + |  |  |
| Sorbitol | + |  |  |
| Sucrose | + |  |  |
| Salicin | + |  |  |
| Trehalose | + |  |  |

+: positive; -:negative

**TABLE S2 |** Identification of virulence factors in the genome of the *Lacticaseibacillus rhamnosus* SC07 using the Virulence Factor Database.

| **Gene_ID** | **VF**  **Category** | **VF**  **Name** | **Function** | **VF_Gene**  **Name** | **VF_Gene**  **Description** | **Identity**  **(%)** | **Evalue** | **Gene**  **Coverage**  **(%)** | **VF_Gene**  **Coverage**  **(%)** |
| --- | --- | --- | --- | --- | --- | --- | --- | --- | --- |
| orf01751 | Immune modulation | Capsule | Resistant to complement deposition and masks cell wall-associated complement from being recognized by the complement receptors on phagocytes | rfbA | glucose-1-phosphate thymidylyltransferase RfbA | 74.4 | 1.04e-159 | 99.7 | 100 |
| orf01753 | Immune modulation | Capsule | Resistant to complement deposition and masks cell wall-associated complement from being recognized by the complement receptors on phagocytes | rfbB | dTDP-glucose 4,6-dehydratase | 78.3 | 4.36e-207 | 98.8 | 96.8 |
| orf02100 | Regulation | LisR/LisK | -- | lisR | two-component response regulator | 78.1 | 5.84e-126 | 100 | 100 |
| orf02699 | Immune modulation | Capsule | Resistant to complement deposition and masks cell wall-associated complement from being recognized by the complement receptors on phagocytes | hasC | UTP--glucose-1-phosphate uridylyltransferase HasC | 70.5 | 4.47e-143 | 96.1 | 97.4 |

**FIGURE S1** | 16S rDNA-based phylogenetic tree and cell morphology (1000×) of the strain SC07.

**FIGURE S2** | Detection of antibiotic resistance genes in the genome of the strain SC07 by in silico screening against the Comprehensive Antibiotic Resistance Database (CARD) database.

**FIGURE S3** | Differential gene expression in the liver transcriptomic analysis. **(A)** and **(B)** Volcano map of differential gene expression. **(C)** The Venn Diagram of differential gene expression. Normal control group (NC), model control group (MC) and oral *Lacticaseibacillus rhamnosus* SC07 group (SC07). For liver transcriptome analysis, n = 8 mice per group.

**FIGURE S4** | The raw image of the full polyvinylidene fluoride membranes in Western blotting analysis. Immunoreactive bands were visualized using the DAB Horseradish Peroxidase Color Development Kit (Beyotime Biotechnology, Shanghai, China). Normal control group (NC), model control group (MC) and oral *Lacticaseibacillus rhamnosus* SC07 group (SC07).
